# An Unusual Case of Catastrophic Antiphospholipid Syndrome in an Elderly Man

**DOI:** 10.7759/cureus.34810

**Published:** 2023-02-09

**Authors:** Kayla Lam, Mohammad Selim

**Affiliations:** 1 Internal Medicine, Creighton University School of Medicine, Omaha, USA

**Keywords:** catastrophic apls, apls, aps, antiphospholipid syndrome, catastrophic aps, catastrophic antiphospholipid antibody syndrome(caps), caps

## Abstract

Antiphospholipid syndrome (APS) is a condition in which the body produces antiphospholipid antibodies, resulting in arterial and venous thrombosis. Catastrophic antiphospholipid syndrome (CAPS) is a rare APS subtype characterized by acute thrombotic microangiography. Antiphospholipid antibodies cause thrombosis through activating and inhibiting properties. CAPS is caused by conditions or factors that trigger the production of antiphospholipid antibodies: genetics that increases the risk of antiphospholipid antibody-associated thrombosis, infection, surgery, medications, and malignancy. We present a unique case of CAPS in a 63-year-old male patient.

## Introduction

Antiphospholipid syndrome (APS) is a condition in which the body produces antiphospholipid antibodies, resulting in arterial and venous thrombosis, most commonly impacting the lower limbs and cerebral arterial circulation [1]. Catastrophic antiphospholipid syndrome (CAPS) is a rare, life-threatening subtype of APS (<1% APS patients), characterized by acute thrombotic microangiography that can result in multi-organ failure in a short period of time [2, 3]. This case study describes an unusual patient with CAPS, which occurs in a 63-year-old male. CAPS has been noted to occur in middle-aged females [2].

## Case presentation

**Figure 1:**
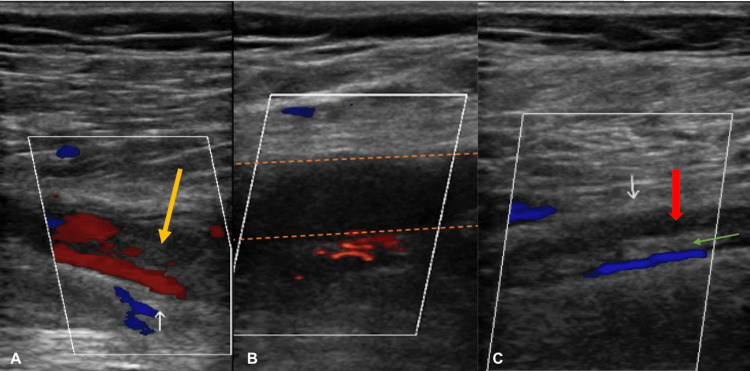
Doppler venous ultrasound demonstrating thromboses in the right (A) femoral, (B) popliteal, and (C) peroneal veins The orange arrow is pointing at the thrombus within the femoral vein. Dashed orange lines outline the popliteal vein. The red arrow is indicating an extensive, active thrombus within the peroneal vein. The green arrow is pointing at an old thrombus within the peroneal vein.

A 63-year-old male with a history of hypertension presented to the emergency department (ED) with a two-day history of increasingly worse shortness of breath (SOB) and chest tightness. The patient’s SOB was minimal at rest but aggravated with exertion. Lung sounds were clear bilaterally, albeit slightly reduced. The skin was noted to be pale. The serum troponin level was increased from 66 ng/L to 150 ng/L over the course of 45 hours (male normal range ≤77 ng/L). Doppler ultrasound showed deep vein thromboses (DVTs) in the right femoral, popliteal, and peroneal veins (Figures 1A-1C).

**Figure 2:**
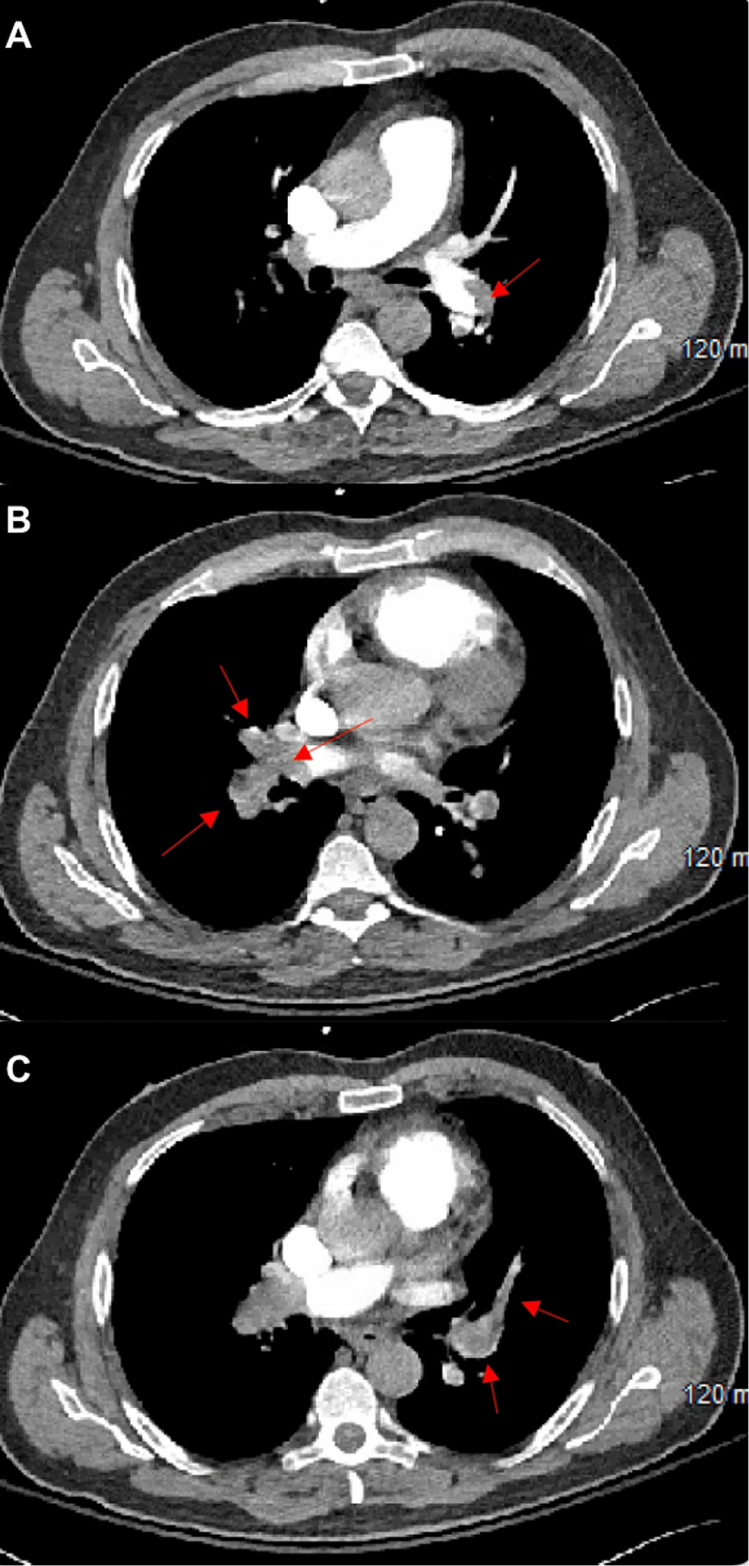
CT chest angiogram demonstrating pulmonary emboli in (A) the left main pulmonary artery, (B) the right main pulmonary artery and right lobar branches, and (C) the left lobar and segmental branches. Red arrows indicate pulmonary emboli.

The electrocardiogram (EKG) was unremarkable, and the complete blood count (CBC) showed a low platelet count of 35,000/uL (no previous history of thrombocytopenia), serum creatinine was 2.2 (previous levels for the past two years were 1.41-1.47 mg/dL), blood urea nitrogen (BUN) was 33, and the congestive heart failure (CHF) peptide was 10,000 pg/mL. The echocardiogram showed severe right ventricular (RV) dilatation and reduced RV function. The interventricular septum flattened during diastole. A computed tomography (CT) with angiogram was ordered and showed sub-massive bilateral pulmonary embolism (PE), spanning from the right and left main pulmonary arteries into the lobar and segmental branches and indicating a cause for the right heart failure (Figure 2A-2C). 

Catheter-directed thrombolysis was done to resolve the pulmonary embolism. Serum creatinine levels and BUN were repeated four hours later and found to have decreased to 1.6 and 28, respectively. On the fourth day of admission, the platelet count decreased to 27,000/uL, and there were no signs of hemolysis. The patient was then switched from low-dose heparin to apixaban. Due to the combination of acute thrombosis and thrombocytopenia, APS was suspected. Because the patient was in post-catheter-directed thrombolysis status, the drop in platelet count could not be attributed to deep vein thrombosis (DVT) or pulmonary embolism (PE). Tests for antiphospholipid antibodies, anticardiolipin, and anti-BGP-1 came back positive. Disseminated intravascular coagulation (DIC) and heparin-induced thrombocytopenia (HIT) were deemed less likely due to a low score based on the International Society on Thrombosis and Haemostasis (ISTH) criteria for disseminated intravascular coagulation (DIC) and based on the low 4T-score for the HIT pretest probability [4, 5]. Consequently, HIT antibodies were not measured. On day five of admission, the patient was switched over to warfarin. The patient was discharged on the sixth day of admission with prednisone and warfarin prescribed. Six weeks later, he was still positive for antiphospholipid antibodies. As a result, this patient was diagnosed with antiphospholipid syndrome and further suspected of having catastrophic APS.

## Discussion

Antiphospholipid syndrome has been under-researched in terms of epidemiology, and the first population-based study on the epidemiology of APS itself was just recently published in 2019 [6]. Antiphospholipid syndrome presents with arterial and venous thrombosis, most commonly impacting the lower limbs and cerebral arterial circulation [1]. Less than 1% of patients present with catastrophic antiphospholipid syndrome, a rare, life-threatening subtype of APS characterized by acute thrombotic microangiography that can result in multiorgan failure in a short period of time [2, 3]. The antiphospholipid antibodies cause thrombosis through activating and inhibiting properties. For activation, the antibodies target endothelial cells, platelets, immune cells, and complement, inducing inflammation and thrombosis. The antibodies also inhibit anticoagulants and deter fibrinolysis. CAPS is caused by conditions or factors that trigger the production of antiphospholipid antibodies: genetics that increase the risk of antiphospholipid antibody-associated thrombosis (ex: coagulation factor mutations), infection (ex: Borrelia burgdorferi, treponema, HIV, Leptospira), surgery, medications (ex: chlorpromazine, procainamide, quinidine, phenytoin), and malignancy [1,2,3,7]. Furthermore, CAPS is often associated with autoimmune disorders, with systemic lupus erythematosus (SLE) being the most common (40%) [2]. Regarding the patient population, 72% of CAPS patients are women, ranging in age from 11 to 60 years old, with a mean age of 37 [2]. In this case, the patient was a man, on the far upper end of the age spectrum, at the age of 63, which is unusual in terms of both age and sex.

As for the criteria to diagnose CAPS, all four of the following requirements must be fulfilled for a definitive diagnosis: (1) involvement of at least three organs; (2) development of manifestations in less than a week; (3) histological evidence of intravascular thrombosis; and (4) presence of antiphospholipid antibodies on two occasions, six weeks apart [2]. CAPS is probable but not definitive if any subset of the criteria occurs in the following manner: 1, 2, 4; 1, 3, 4, but the third event occurs between a week and a month even with anticoagulation; 1-4, but only two organs are affected; and 1-4, but the second antiphospholipid antibodies assay could not happen at least six weeks after the first due to patient death [2]. In our case, the patient fulfilled one, but only two organs: the heart (elevated serum troponin levels and right heart strain) and kidneys (elevated creatinine and BUN until they dropped post-thrombolysis) were affected. He also fulfilled requirements three and four. Thus, the suspicion of CAPS was confirmed.

Regarding treating CAPS, the consensus is to employ anticoagulants and corticosteroids in all patients [8]. While there are no randomized-controlled trials (RCTs) on specific types of anticoagulants in CAPS specifically due to low prevalence, there have been several recent studies on APS in general showing that warfarin prevents anticoagulation more efficaciously than direct oral anticoagulants (DOACs) and that DOACs increase the risk of thromboses compared to warfarin [8, 9, 10]. Hence, the patient was switched from apixaban to warfarin upon the suspected diagnosis of CAPS.

## Conclusions

Based on this unusual instance of CAPS, there is a need to keep an open mind when diagnosing and not be limited by the common patient presentation. Considering that CAPS is not as frequently seen as typical APS and yet is also life-threatening, this unusual appearance of CAPS in a more elderly male rather than the typical middle-aged female calls for the need to screen all patients with evidence of intravascular thrombosis for CAPS in order to treat CAPS earlier and reduce mortality from this disease. Additional studies should be done to further identify differences in risks between CAPS and generic APS to help prompt earlier prevention or intervention, especially since this patient had no prior medical history besides hypertension.
